# High-amplitude network co-fluctuations linked to variation in hormone concentrations over the menstrual cycle

**DOI:** 10.1162/netn_a_00307

**Published:** 2023-10-01

**Authors:** Sarah Greenwell, Joshua Faskowitz, Laura Pritschet, Tyler Santander, Emily G. Jacobs, Richard F. Betzel

**Affiliations:** Department of Psychological and Brain Sciences, Indiana University, Bloomington, IN, USA; Program in Neurosciences, Indiana University, Bloomington, IN, USA; Department of Psychological and Brain Sciences, University of California, Santa Barbara, Santa Barbara, CA, USA; Neuroscience Research Institute, University of California, Santa Barbara, Santa Barbara, CA, USA; Cognitive Science Program, Indiana University, Bloomington, IN, USA; Network Science Institute, Indiana University, Bloomington, IN, USA

**Keywords:** Edge-centric, Functional connectivity, Time-varying networks

## Abstract

Many studies have shown that the human endocrine system modulates brain function, reporting associations between fluctuations in hormone concentrations and brain connectivity. However, how hormonal fluctuations impact fast changes in brain network organization over short timescales remains unknown. Here, we leverage a recently proposed framework for modeling co-fluctuations between the activity of pairs of brain regions at a framewise timescale. In previous studies we showed that time points corresponding to high-amplitude co-fluctuations disproportionately contributed to the time-averaged functional connectivity pattern and that these co-fluctuation patterns could be clustered into a low-dimensional set of recurring “states.” Here, we assessed the relationship between these network states and quotidian variation in hormone concentrations. Specifically, we were interested in whether the frequency with which network states occurred was related to hormone concentration. We addressed this question using a dense-sampling dataset (*N* = 1 brain). In this dataset, a single individual was sampled over the course of two endocrine states: a natural menstrual cycle and while the subject underwent selective progesterone suppression via oral hormonal contraceptives. During each cycle, the subject underwent 30 daily resting-state fMRI scans and blood draws. Our analysis of the imaging data revealed two repeating network states. We found that the frequency with which state 1 occurred in scan sessions was significantly correlated with follicle-stimulating and luteinizing hormone concentrations. We also constructed representative networks for each scan session using only “event frames”—those time points when an event was determined to have occurred. We found that the weights of specific subsets of functional connections were robustly correlated with fluctuations in the concentration of not only luteinizing and follicle-stimulating hormones, but also progesterone and estradiol.

## Author Summary

We study a dense sampling dataset of one brain (*N* = 1) imaged across two complete menstrual cycles (60 scan sessions). We identify network states—high-amplitude patterns of time-varying connectivity that reoccur across scan sessions—and show that the frequency with which states occur is linked to endogenous fluctuations in follicle-stimulating and luteinizing hormones. We further show that the weights of scan-specific and whole-brain co-fluctuation patterns are broadly associated with hormone fluctuations.

## INTRODUCTION

The human brain is a complex network composed of structurally connected neural elements that help shape brain activity and give rise to widespread patterns of functional coupling (Hermundstad et al., 2013; Sporns, Tononi, & Kötter, 2005). The organization and topology of these structural and functional networks can be interrogated using tools from network science (Bullmore & Sporns, 2009), revealing organizing principles including short processing paths (Bassett & Bullmore, 2006), hubs and rich clubs (Hagmann et al., 2008; van den Heuvel & Sporns, 2011), modular structure (Sporns & Betzel, 2016), and cost-efficient spatial embedding (Bullmore & Sporns, 2012).

Recent work has shown that whole-brain patterns of functional coupling between brain regions vary over short timescales (Allen et al., 2014; Barttfeld et al., 2015; R. F. Betzel, Fukushima, He, Zuo, & Sporns, 2016). To reconstruct changes in network structure over time, most studies use sliding-window analyses (Hindriks et al., 2016; Preti, Bolton, & Van De Ville, 2017). In this approach, a functional network is estimated using only those samples that fall within a window of some fixed duration. The window is then advanced a certain number of frames, resulting in a time series of functional networks. Although applied widely, this approach has a number of drawbacks. Namely, it forces the user to specify parameters for window length and amount of overlap between successive windows. The windowing procedure, itself, also makes it impossible to precisely localize a network state to a specific moment in time and resolve changes in network structure over short timescales (Hindriks et al., 2016; Leonardi & Van De Ville, 2015; Liégeois, Laumann, Snyder, Zhou, & Yeo, 2017; Lurie et al., 2020).

Recently, we proposed edge time series (ETS) as a method for decomposing functional networks into time-varying components (Esfahlani et al., 2020; Faskowitz, Esfahlani, Jo, Sporns, & Betzel, 2020; Sporns, Faskowitz, Teixeira, Cutts, & Betzel, 2021). This approach helps address some of the limitations of sliding-window analyses, in that it is parameter-free and can resolve changes in network structure at a framewise timescale. In previous studies, we used this method to show that fast network dynamics are not smooth, but rather are bursty, identifying long periods of quiescence punctuated by brief, network-wide, high-amplitude events (R. F. Betzel, Cutts, Greenwell, Faskowitz, & Sporns, 2022; Esfahlani et al., 2020; Pope, Fukushima, Betzel, & Sporns, 2021). These events are of particular interest, as time-averaged functional networks can be accurately reconstructed from a very small number of events. We also found that the patterns of co-fluctuation expressed during events contain disproportionate amounts of information about an individual and that events can improve brain-behavior correlations. Despite their apparent utility, the application of edge time series for linking brain dynamics to cognitive, clinical, or physiological phenomena has been limited (Cooper, Kurkela, Davis, & Ritchey, 2021; Esfahlani et al., 2022; Sorrentino et al., 2021; Tanner et al., 2022).

Edge time series are well situated for investigating relationships between brain connectivity and physiological variables that also fluctuate over short timescales. A good example is the human menstrual cycle, which is typified by variations in the sex steroid hormones estradiol—the major form of estrogen in most mammals—and progesterone and gonadotropins follicle-stimulating hormone (FSH) and luteinizing hormone (LH). Briefly, during the follicular phase of the menstrual cycle estradiol concentrations rise, prompting the growth of the uterine lining. Immediately prior to ovulation, FSH encourages an immature follicle to complete its development into an egg for release from the ovaries. LH promotes the release of the egg into the fallopian tubes, followed by an increase in progesterone during the luteal phase, during which the uterine lining thickens, creating a favorable environment for the egg to be preserved. These hormones exhibit neuromodulatory effects and have been associated with variation in brain connectivity, neural structure, and brain activity (Beltz & Moser, 2020; Berman et al., 1997; Jacobs & D’Esposito, 2011; McEwen, Akama, Spencer-Segal, Milner, & Waters, 2012; Taxier, Gross, & Frick, 2020). However, most previous work has involved sparse cross-sectional studies that capture one or more time points across the menstrual cycle (Hidalgo-Lopez et al., 2020; Pletzer, Harris, Scheuringer, & Hidalgo-Lopez, 2019; Weis, Hodgetts, & Hausmann, 2019) or cross-sectional study designs with incomplete sampling of participants’ cycles and comprised of heterogeneous cohorts of individuals (Dubol et al., 2021).

Recently, a series of “dense sampling” studies have characterized quotidian variation in these hormones and their relationships with functional connectivity and network community structure (Fitzgerald, Pritschet, Santander, Grafton, & Jacobs, 2020; Mueller et al., 2021; Pritschet et al., 2020; Taylor et al., 2020). The design of these studies parallels that of other recent dense sampling studies (Braga & Buckner, 2017; Gordon et al., 2017; Laumann et al., 2015), acquiring data from, in this case, a single individual over the course of two complete menstrual cycles, one in which the participant was naturally cycling and another while the same participant was placed on oral hormonal contraception that selectively suppressed progesterone concentrations by ≈97% (referred to as Studies 1 and 2) (Pritschet, Taylor, Santander, & Jacobs, 2021). Oral contraceptives are estimated to be used by over 100 million women of reproductive age worldwide (Christin-Maitre, 2013). “The pill” generally contains synthetic analogs of combined estrogen and progesterone or only progesterone. The pill inhibits the spike in gonadotropins that induces ovulation, thereby preventing pregnancy. This user followed the typical pattern of intake, taking the active hormonal pill for 3 weeks before taking a nonhormonal ‘sugar pill’ during the fourth and final week. Previous studies using these datasets have identified spatially diffuse increases in functional connectivity and modular flexibility coincident with peaks in estradiol; progesterone, by contrast, was predominantly associated with reductions in connectivity (Mueller et al., 2021; Pritschet et al., 2020). However, the dynamic underpinnings of these associations have not been fully explored.

Here, we analyze these two fMRI datasets, one during the subject’s natural cycle and the other while the subject was placed on an oral contraceptive regimen. We do not compare between hormone states in this study. Instead, we pool data from both states in our analyses. By pooling data from a synthetically altered hormonal state and a natural hormonal state in the same individual, we can examine graded and continuous relationships between brain networks and the concentrations of individual hormones. In more detail, we follow the analysis pipeline from R. F. Betzel, Cutts, et al. (2022), in which we use edge time series to detect events from functional magnetic resonance imaging (fMRI) data and cluster whole-brain co-fluctuation patterns into a low-dimensional set of states. Our primary aim is to assess whether properties of these states can be statistically linked to hormone fluctuations and, if so, whether those associations exhibit specificity such that they implicate specific subsets of hormones and not others. In agreement with previous studies, we find that events can be subdivided into two distinct clusters. In particular, we show that the frequency with which the first cluster appears in a given scan is strongly correlated with quotidian variation in both FSH and LH. Furthermore, we show that day-to-day variation in the edge- and system-level configuration of the first cluster is broadly associated with both FSH and LH, as well as progesterone and estradiol. Collectively, our results establish a new link between high-amplitude, network-level events and the human endocrine system, opening up new avenues for exploring the dynamic interplay between brain-hormone relationships.

## RESULTS

### Clustering High-Amplitude Co-Fluctuations Reveal Distinct Patterns of Connectivity

**Figure 1.**
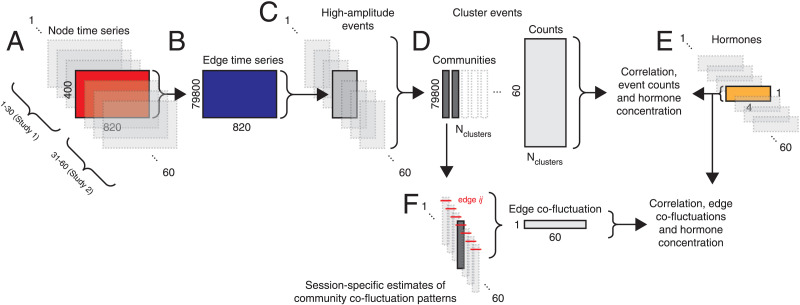
Analysis pipeline. (A) After preprocessing, we obtained parcellated regional time series from 60 scans (spanning two experiments). (B) For a given scan, we transformed node time series into edge time series following Esfahlani et al. (2020). (C) Next, we detected high-amplitude events in each scan and for each event extracted its representative pattern (the frame with the greatest amplitude). In general, we obtained a different number of events per scan. (D) We aggregated event patterns from all scans and collectively clustered them using modularity maximization. This procedure resulted in multiple community centroids (we analyze the two largest) and a count of how many times a given community appeared on a given scan session. (E) In parallel, we analyzed hormone data that were collected concurrent with each scan session. Our principal aim was to link features of communities (brain states) with hormone data. (F) In addition, we reconstructed estimates of communities for each of the 60 scan sessions and, for each edge, computed the correlation of its co-fluctuation across sessions with hormone concentrations.

Recent methodological advances have made it possible to track rapid fluctuations in functional network architecture, revealing the presence of “events”—short-lived and high-amplitude patterns of network-wide fluctuations (Esfahlani et al., 2020; Faskowitz et al., 2020; Sporns et al., 2021). The results of previous studies suggested that events are low dimensional, such that a small repertoire of patterns are reiterated (R. F. Betzel, Cutts, et al., 2022; Pope et al., 2021). Here, we test whether this was also the case in an independently acquired dataset of a single individual. Figure 1 depicts a schematic of our general analysis pipeline.

To detect and assess whether there were repeated patterns of high-amplitude events, we used simple statistical procedure to identify sequences of temporally contiguous frames whose root summed square (RSS) exceeded that of a null model in which regions’ activity time courses were randomized (circular shifts). Note that this null model approximately preserves local properties of regional activity, but destroys brain-wide correlation structure. Thus, this model is not, strictly speaking, a test of dynamics in co-fluctuations. We retained only those sequences that were not obviously coincident with periods of excessive in-scanner movement, that is, did not include a high-motion frame and were at least two frames away from any high-motion frame. For each such sequence, we extracted the co-fluctuations expressed during the frame with the highest RSS value as a representative peak. In total, we detected 899 motion-free events that occurred during low-motion intervals (14.98 ± 5.27 events per scan session).

**Figure 2.**
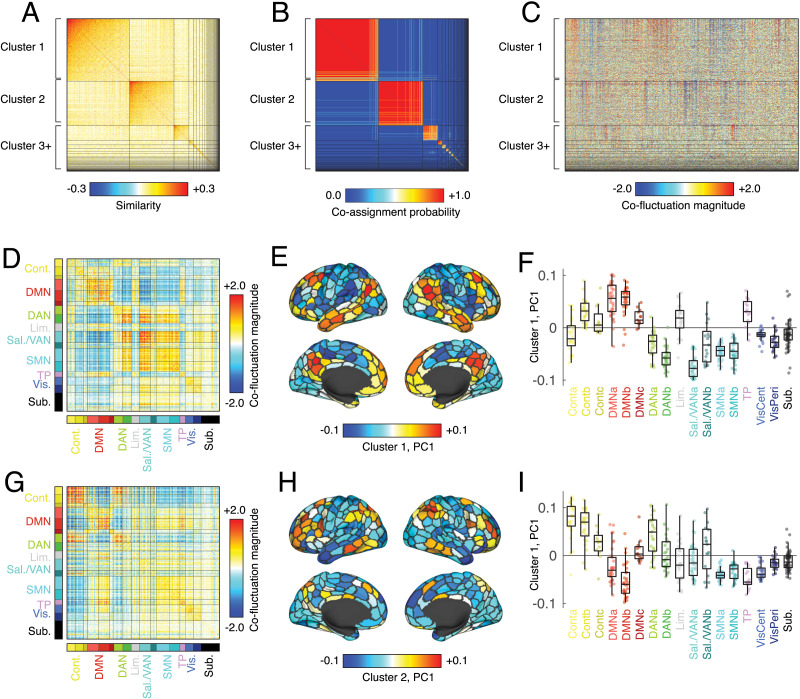
Modularity maximization and network states. We used an event detection algorithm to identify instances of “high-amplitude” co-fluctuations, extracting 899 brain-wide patterns. Each putative event was represented as a region-by-region co-fluctuation matrix. We vectorized each matrix by extracting its upper triangle elements and calculated the spatial similarity (Pearson correlation) between all pairs of patterns. This resulted in a 899 × 899 similarity matrix. We used this matrix as input to a clustering algorithm (modularity maximization) to detect groups or “communities” of mutually similar co-fluctuation patterns. Modularity maximization is nondeterministic, so we repeated the algorithm 1,000 times with random restarts and assembled the partitions into a co-assignment matrix, whose elements counted the fraction of the 1,000 partitions in which any pair of co-fluctuation patterns were assigned to the same community. To resolve variability across runs, we iteratively clustered the co-assignment matrix to obtain “consensus communities.” We refer to each consensus community as a “state.” (A) Similarity matrix, ordered by consensus communities. (B) Community co-assignment matrix ordered by consensus communities. (C) Vectorized co-fluctuation patterns ordered by consensus communities. (D) Mean co-fluctuation matrix for cluster 1, ordered by canonical brain systems. (E) First principal component of the co-fluctuation matrix. (F) Elements of first principal component grouped by brain system. Panels G–I are analogous to D–F but for cluster 2.

To detect putative states, clusters, or communities of events (note that here we use these terms interchangeably), we calculated for each event its whole-brain co-fluctuation pattern—the *N* × *N* matrix whose element {*i*, *j*} was equal to the product of z-scored activity in regions *i* and *j* at the time of the event. We then vectorized the event co-fluctuation patterns and, for all pairs of events, computed their spatial similarity (Pearson correlation), resulting in a 899 × 899 matrix. We then used a variant of modularity maximization to assign each co-fluctuation pattern to a single cluster (see Materials and Methods for details). As in previous studies, we found evidence for two large consensus communities that appeared in most scan sessions and collectively accounted for ≈70.2% of all detected states (Figure 2A). These clusters were highly reproducible across repeated runs of the modularity optimization algorithm (Figure 2B) and divided event patterns into cohesive clusters (Figure 2C). We note that these clusters were also robust to variation in processing pipelines, after splitting the data by experiment (see Supporting Information Figure S2), and after systematically excluding data from individual scans in calculating the correlation (Supporting Information Figure S6). Finally, we also assessed what effect using null distributions of RSS values from different scan sessions had on the detected events. In general, we found a near perfect correspondence, suggesting that event structure is not strongly impacted by quotidian variation in the null distribution (Supporting Information Figure S3).

Interestingly, the spatial patterns of these clusters closely recapitulate those reported in previous studies (R. F. Betzel, Cutts, et al., 2022; Esfahlani et al., 2020). Community 1, for instance, was characterized by opposed fluctuations between regions in the default mode network with those in dorsal and salience/ventral attention networks (Figure 2D). To better understand whether these co-fluctuations were underpinned by a specific mode or pattern of activity, we performed a singular value decomposition of the mean co-fluctuation matrix, revealing, as expected, a node-level pattern characterized by strong fluctuations in default mode regions and opposed fluctuations in attentional and, to some extent, sensorimotor systems (Figure 2E and F). Similarly, the spatial pattern of community 2 was characterized by opposed co-fluctuations of control and dorsal attention regions with the default mode network (Figure 2G–I).

The remaining communities collectively accounted for <30% of all events, with the next most frequent community accounting for 9.8% of all events (but appearing in ≈71.7% of scan sessions). For this reason, we focus on the first two communities for all subsequent analyses. In the Supporting Information we describe the remaining communities in greater detail (Supporting Information Figure S1).

### State Frequency Is Associated With LH and FSH Concentrations

Cluster analysis of co-fluctuation time series revealed the presence of repeating patterns or states. However, the biological relevance of these states remains unclear. In this section, we show that the frequency with which these states appear across scan sessions is robustly related to endogenous variation in the gonadotropins, luteinizing, and follicle-stimulating hormone, but not statistically associated with sex hormones estradiol and progesterone.

**Figure 3.**
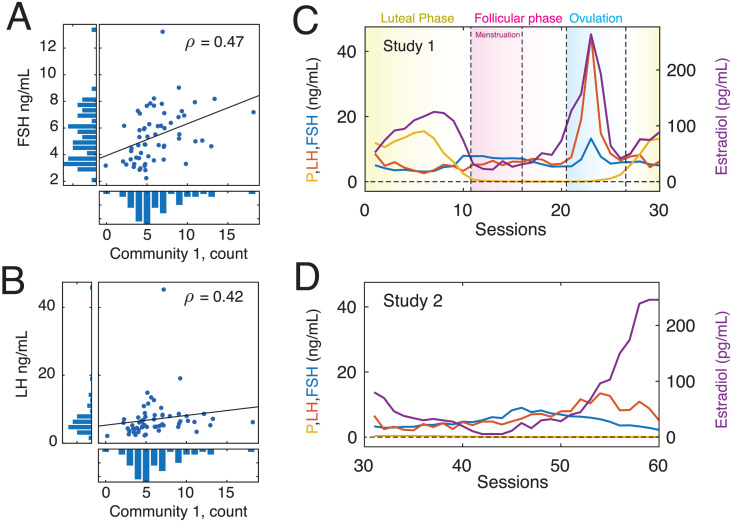
Correlations between state frequency and quotidian variation in gonadotropin concentration. (A) Scatterplot showing concentration of follicle-stimulating hormone across scan sessions versus the frequency with which cluster 1 appeared in a given scan. (B) Scatterplot showing concentration of luteinizing hormone across scan sessions versus the frequency with which cluster 1 appeared in a given scan. Panels C and D show variation of progesterone, estradiol, follicle-stimulating hormone, and luteinizing hormone across the Study 1 and Study 2 datasets.

To link high-amplitude co-fluctuations with quotidian variation in hormone concentration (see Figure 3C and D), we calculated for each scan session the number of times that each community (1 and 2) appeared. We found that, on average, communities 1 and 2 appeared 6.15 ± 3.17 and 4.37 ± 2.57 times (≈41% and ≈29% of all events; maximum values of 18 and 11), respectively. Next, we computed the Spearman rank correlation of these frequencies with hormone concentrations. Note that the rank correlation reduces statistical biases originating from spikes in both luteinizing and follicle-stimulating hormone around ovulation. For all reported correlations we corrected for multiple comparisons by fixing the false discovery rate at *q* = 0.05, resulting in an adjusted critical value of *p*_*adj*_ = 0.0067. We found statistically significant correlations between the frequency of community 1 with both gonadotropic hormones (*ρ*_1,*FSH*_ = 0.47 and *ρ*_1,*LH*_ = 0.42; *p* = 0.0002 and *p* = 0.0007; effect sizes of *d* = 1.07 and *d* = 0.93, Cohen’s *d*). Interestingly, the correlations between community 1 and progesterone (*ρ*_1,*P*_ = −0.33; *p* = 0.007) and community 2 with luteinizing hormone (*ρ*_2,*LH*_ = 0.26; *p* = 0.02; effect sizes of *d* = 0.35 and *d* = 0.27, Cohen’s *d*) were both statistically significant at uncorrected levels, but failed to pass after multiple comparison corrections (see Supporting Information Figure S8). The results described above were obtained after pooling data from two separate experiments to obtain 60 observations in total (30 observations per experiment), improving statistical power. However, the protocols differed between experiments: in one the subject took an oral contraceptive and in the other the subject did not. To address the concern that the results are driven by one of the experiments and not the other or from inappropriately combining dissimilar protocol, we repeated our analyses separately for the two datasets (30 observations each). We find that those results are consistent with what was reported in the main text with no discernible differences between the two datasets, suggesting that combining the two is reasonable (Supporting Information Figure S4). We also compared hormone concentrations between experiments and found that, of the four hormones reported here, only progesterone exhibited significant differences (Supporting Information Figure S5). Note that we also verify that results are not dependent on processing pipeline, replicating our main effects using a pipeline in which global signal regression was not included (Supporting Information Figure S9).

Notably, however, we found that both FSH and LH were correlated with total event count (the number of events detected in a given scan session; *ρ*_*FSH*,*total*_ = 0.21 and *ρ*_*LH*,*total*_ = 0.33) and that when we expressed the event count for state 1 as a proportion of the event count for each session, the effect was in the same direction but attenuated (*ρ*_*FSH*,*prop*_ = 0.29 and *ρ*_*LH*,*prop*_ = 0.09). These observations suggest that the relationship of hormone concentrations with event structure is dually global—linked to the overall event count on any given scan session—but allows for local associations with the frequency and proportion of specific co-fluctuation states.

Collectively, these results suggest that endogenous and exogenously induced changes in gonadotropic hormone concentrations are related to the expression of distinct brain network states. These findings address our stated aim of linking brain states defined based on edge time series with hormonal fluctuations. Further, these findings suggest some specificity at both the brain and endocrine levels, implicating only a single brain state and a pair of hormones (FSH and LH, but not estradiol or progesterone). Finally, these findings also suggest that hormones may have a role in modulating variation in high-amplitude network-level co-fluctuations.

### Quotidian Variation in Edge-Level Co-Fluctuations and Hormone Concentrations Are Correlated

In the previous section, we demonstrated that day-to-day variation in gonadotropin concentration was linked to the frequency with which particular high-amplitude “states” are expressed. In that analysis, any co-fluctuation pattern assigned a given community label was treated as a recurrence of the same state. In reality, however, the whole-brain patterns classified by these state motifs exhibited variability between days and even within instances during the same scan. Here, we demonstrate that within-state variation in edge co-fluctuation magnitude is linked not only with gonadotropin concentration, but also with the concentrations of sex hormones estradiol and progesterone.

To link edge-level co-fluctuations with hormones, we needed estimates of communities 1 and 2 for each scan session. To do this, we identified all co-fluctuation patterns assigned to a given community on each scan session and averaged those. Note that in the case of community 1, there was one scan in which it never appeared; in the case of community 2, there were two. We omitted these scans from all analyses carried out in this section. For each of the remaining scans, we created a representative version of community 1 and 2 centroids by averaging all co-fluctuation assigned to that community. We also kept track of the number of samples used to compute the representative pattern (i.e., the number of times that a given state was present in each scan) and after aggregating across all scans, regressed out this number from each node pair, retaining the residuals and calculating their Spearman rank correlation with the concentrations of progesterone, estradiol, luteinizing hormone, and follicle-stimulating hormone.

**Figure 4.**
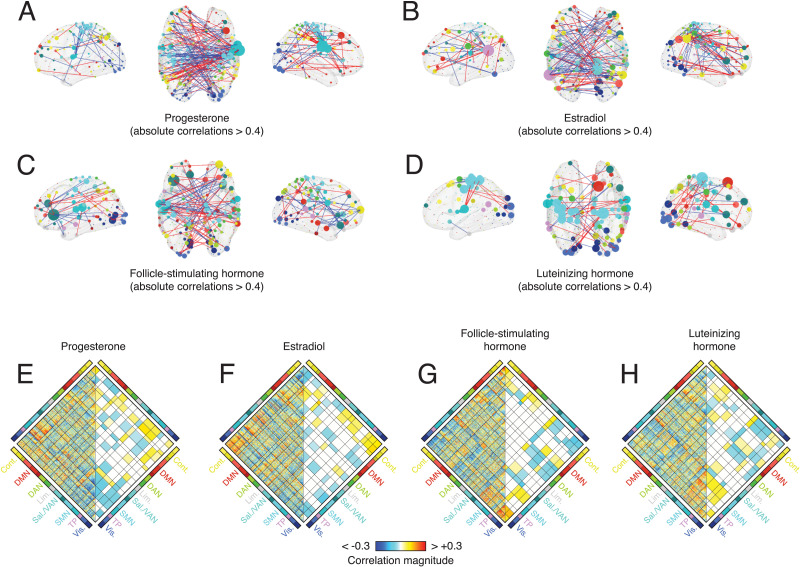
Edge- and system-level correlations with hormone concentration. We calculated the correlation of hormone concentrations with edge-level co-fluctuation magnitudes for cluster 1. This procedure resulted in a correlation coefficient at every edge (*N*_*edges*_ = 400 × 399/2 = 79,800) and separately for every hormone. Rather than perform statistical tests at the level of edges, we performed tests at the level of systems. That is, for every pair of systems, we calculated the mean correlation of all edges that fall between those two systems and compare that value against what would be expected by chance. This procedure results in *N*_*systempairs*_ = 136 unique pairs of systems and offers greater statistical power due to fewer independent tests. We corrected for multiple comparisons by fixing the accepted false discovery rate (the probability of falsely detecting an effect when no such effect exists) to 5% and adjusted the critical *p* value to obtain this accepted rate. Thus, for every pair of systems and for every hormone, we were able to assess whether the mean correlation of edges between those systems was statistically stronger than expected. These results are shown in panels E–H. On the left-hand side of each diamond plot are the raw, uncorrected edge-level correlations. On the right-hand side are system-level correlations that were greater than expected. System pairs that did not pass statistical testing are shown in white. The matrices in panels E–H are not anatomically localized and may be difficult to interpret. To provide some intuition and to anatomically ground these effects, in panels A–D, we show only the strongest edge-level correlations projected back into anatomical space. In each panel, node size is proportional to the mean correlation magnitude of a node’s edges. Node color was determined by brain system. Edge color denotes positive (red) and blue (negative) correlations. Note, however, that panels A–D are for provided for the sake of visualization and intuition only. The statistical tests and their outcomes are shown in panels E–H.

Mass univariate edge-level analyses can lack statistical power to resolve certain effects. Here, for instance, we performed 79,800 tests (the number of edges) with the aim of identifying those that pass a criterion for statistical significance (for visualization only, we show the edges with the strongest positive and negative correlations embedded in anatomical space in Figure 4A–D). An alternative strategy is to perform statistical testing at the level of brain systems or communities. Because nodes are aggregated by community, this approach necessarily limits one’s ability to resolve focal, regional effects. However, because the number of comparisons is reduced by an order of magnitude or more, statistical power increases.

Here, we aggregated edge-level correlations by brain system (Schaefer et al., 2018), transforming a correlation matrix of dimensions *ρ*_*region*_ ∈ [400 × 400] into a *ρ*_*system*_ ∈ [16 × 16] matrix. The elements of *ρ*_*system*_ represented the mean correlation coefficients between all pairs of regions assigned to any two systems. We repeated this procedure for all four hormones (Figure 4E–F), yielding four system-level correlation maps. To identify significant correlations, we repeated the aggregation procedure after randomly permuting system labels using a “spin” test to approximately preserve spatial dependencies between regions (1,000 permutations) (Váša et al., 2018).

**Figure 5.**
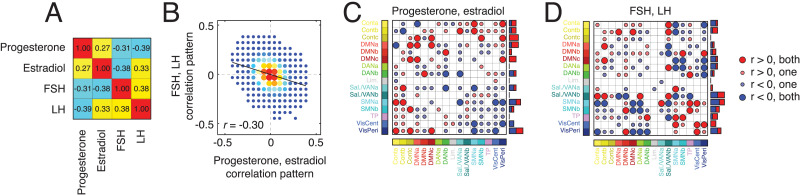
Similarity of brain-wide correlation patterns across hormones. In the main text we described a procedure in which we calculated the correlation of hormone concentration with the co-fluctuation magnitude between every pair of nodes (edges). This procedure resulted in four node × node matrices of correlation coefficients (one for each of the four hormones studied here). (A) Similarity of correlation patterns between pairs of hormones. (B) Plotting hormones against each other yields dense scatterplots that are difficult to interpret. Here, we present the same data as a two-dimensional histogram (by binning *x* and *y* axes and counting the number of points that fall within each bin). Here, the two-dimensional histogram depicts the similarity of correlation patterns from combined sex hormones (progesterone + estradiol) and gonadotropins (FSH + LH). The color is linearly proportional to the number of points in each bin, with brighter colors corresponding to larger values. (C) To further examine similarities and differences between the correlation patterns, we performed a concordance analysis. Specifically, we identified pairs of brain systems in which the mean correlation was statistically greater/less than that of a permutation based null model. We further examined similarities in sex and gonadotropic hormones separately. For every pair of brain systems, there were five possible outcomes: both exhibited significant positive correlations, both exhibited significant negative correlations, one or the other exhibited a significant positive correlation while the other did not, one or the other exhibited a significant negative correlation while the other did not, and neither exhibited a significant correlation. In panel C, we plot the outcomes of this analysis for sex hormones (progesterone and estradiol). Large and small circles indicate high and low levels of concordance. Red and blue colors indicate positive and negative correlations, respectively. The bar plot to the right of the matrix is a count of the total number of high concordance interactions in which a given system interacts. Panel D depicts analogous information for combined gonadotropins (FSH + LH).

In general, we found that the regional correlation patterns for sex hormones were similar to one another (*r*_*P*,*E*_ = 0.27; *p* < 0.05). The same was true for gonadotropins (*r*_*FSH*,*LH*_ = 0.38; *p* < 0.05) (Figure 5A). Due to their similarity and for ease of description, we combined system-level correlation patterns for progesterone with estradiol and FSH with LH, focusing on shared effects (these combined patterns are anticorrelated with one another; *p* < 0.05; Figure 5B). To summarize shared effects, we classified every pair of systems based on the concordance of correlation patterns. That is, whether both, one, or neither hormones exhibited effects in the same direction (false discovery rate fixed at *q* = 0.05 resulting in adjusted critical value of *p*_*adj*_ = 0.0036; Figure 5C and D).

In both cases, we found evidence of broad, brain-wide constellations formed by significant system-level correlations. In the case of sex steroid hormones, correlations involving the control and default mode networks tended to be positive, while correlations involving attention, somatomotor, and visual network tended to be negative (Figure 5C). On the other hand, in the case of gonadotropins, positive and negative correlations were more uniformly distributed across brain systems, with salience/ventral attention, somatomotor, and visual networks ranking among the systems with the greatest overall number of significant correlations (Figure 5D).

We also repeated this analysis using daily estimates of edge-level co-fluctuations for community 2, resulting in four correlation maps (see Supporting Information Figure S7). Overall, we find that LH and FSH exhibit similar whole-brain, edge-level correlation patterns (*r*_*FSH*,*LH*_ = 0.28) but that progesterone and estradiol are largely dissimilar (*r*_*P*,*E*_ = 0.06). Interestingly, LH and estradiol also exhibit strong correlations (*r*_*LH*,*E*_ = 0.49) while the relationship is reversed with FSH and estradiol (*r*_*FSH*,*E*_ = −0.35). As with community 1, the edge level correlations did not clearly favor any particular set of systems; instead, they implicated a constellation of brain areas and systems. In the case of progesterone, some of the strongest correlations involved regions in the default mode and salience/ventral attention networks. Estradiol fluctuations, on the other hand, exhibited strong correlations with edges incident upon visual and control network nodes. In the case of gonadotropins, there was less consensus as to which specific systems were implicated, as the correlation patterns were more diffuse and broadly distributed. However, control, somatomotor, and visual networks appear particular prominent.

Here, we study brain-hormone correlations using cluster centroids from edge time series. An important question is whether these correlational patterns are similar to those obtained in previous studies using FC instead of centroids. Accordingly, we repeated our correlational analysis, substituting static FC matrices for each scan session for the cluster centroids and obtaining matrices of edge-level correlations. We then computed the similarity of these correlation patterns with those obtained using cluster centroids (similarity is measured as the Spearman correlation of upper triangle elements). In general, we find a broad correspondence between the two approaches (mean correlation of *ρ* = 0.30 ± 0.06). However, these results also indicate that the majority of variance remains unexplained, suggesting that the cluster centroids and FC analyses may reveal distinct brain-hormone relationships at the edge level.

Collectively, these findings suggest that the co-fluctuation between specific pairs of brain regions are associated with concentrations of sex steroid hormones as well as gonadotropins. As in the previous section, these results strengthen the link between features of edge time series, which disclose fast fluctuations in coactivity, and hormonal fluctuations. These relationships are expressed through distributed, brain-wide constellations of edges linking together many brain systems.

## DISCUSSION

Here, we aimed to link fast fluctuations in coactivity with hormonal fluctuations and to assess the specificity of those effects. To this end, we built upon previous analyses of a dense-sampling dataset in which a single participant underwent daily MRI scans and serological sampling over the course of two full menstrual cycles (Fitzgerald et al., 2020; Mueller et al., 2021; Pritschet et al., 2020; Taylor et al., 2020). Leveraging “edge time series” (Esfahlani et al., 2020; Faskowitz et al., 2020), we detected high-amplitude events in each scan session, clustering them into two large communities. The first community reflects opposed activation of default mode and control regions with attentional and sensorimotor regions. We found that the frequency with which it appears across scan sessions was strongly coupled to quotidian fluctuations in the gonadotropic hormones, follicle-stimulating hormone and luteinizing hormone. We find that variability in the scan-specific co-fluctuation pattern of community 1 was linked to all four hormones at the level of connections. Our work sets the stage for future studies to investigate relationships between fast fluctuations in network organization and hormones.

### High-Amplitude Co-Fluctuations Are Linked to Endocrine System

Previous applications of edge time series analysis to functional imaging data have reported brief, intermittent, and high-amplitude “events” (R. F. Betzel, Cutts, et al., 2022; Esfahlani et al., 2020, 2022; Pope et al., 2021; Sporns et al., 2021). These studies have shown that the co-fluctuation patterns expressed during events contribute disproportionately to the time-averaged pattern of FC, are subject-specific, can be clustered into a small number of putative “states,” and strengthen brain-behavior associations. For instance, in Esfahlani et al. (2020), the authors show that the overall magnitude of brain-behavior correlations increases when the brain measures are derived for high-amplitude co-fluctuations compared to low-amplitude. However, the physiological underpinnings of high-amplitude co-fluctuations remain unclear.

Here, we present evidence that both the frequency with which high-amplitude states occur as well as their topology are strongly correlated with day-to-day fluctuations in hormone concentration. The findings suggest that hormones may have a role in modulating variation in high-amplitude network-level co-fluctuations. While this observation is in line with previous studies that have reported links between reproductive hormones and both brain activity (Berman et al., 1997; Jacobs & D’Esposito, 2011) and functional connectivity (Arélin et al., 2015; Dan et al., 2020; Fitzgerald et al., 2020; Hidalgo-Lopez, Zeidman, Harris, Razi, & Pletzer, 2021; Liparoti et al., 2021; Lisofsky et al., 2015; Petersen, Kilpatrick, Goharzad, & Cahill, 2014; Pritschet et al., 2020; Weis et al., 2019), our results extend this link to ultrafast network dynamics. Changes in network structure at this timescale have been relatively unexplored in previous studies due to their cross-sectional study design and low temporal resolution of sliding-window methods for estimating time-varying functional connectivity (Dubol et al., 2021) (although, see Mueller et al., 2021).

While the design of our study is not well-suited to support claims about causality, we speculate that by modulating receptor density during the menstrual cycle, hormones may shift the sensitivity of neural circuits, yielding brain states that are increasingly excitable and more likely to produce high-amplitude events (Li et al., 2019; Liu & Herbison, 2013; Shine, Aburn, Breakspear, & Poldrack, 2018). Indeed, recent studies have demonstrated that the sex hormones estrogen and progesterone differentially influence the density of steroid hormone receptors across the brain throughout the menstrual cycle (McEwen & Milner, 2017). Moreover, this view aligns with the nonlinear and nonstationary contribution of luteinizing hormone to hormone dynamics across the menstrual cycle; large surges in LH near ovulation disrupt the phasic coupling of sex steroid and gonadotropic hormones (Dubol et al., 2021), suggesting that structural changes caused by acute changes in hormone concentration, such as receptor density, influence network connectivity in addition to the direct effects of hormones on cells. Sex hormones are intrinsically involved in micro-level structural changes as well, given that estrogen- and progesterone-mediated changes in subcortical receptor density induce the release of gonadotropin-releasing hormone to induce luteinizing hormone spike (Sinchak, Mohr, & Micevych, 2020).

Another possible explanation for why fluctuations in reproductive hormones are associated with brain network dynamics concerns their role as neurotransmitters. Previous studies have established links between neurotransmitters and functional connectivity, demonstrating that regional variation in the concentration of neurotransmitter-related neurons modulate large-scale brain activity, shaping patterns of connectivity and the spontaneous emergence of resting-state networks (Dang, O’Neil, & Jagust, 2012). For example, dopamine and serotonin oppositionally influence anticorrelations between large-scale networks, such that dopamine is associated with increases and decreases in functional connectivity of the somatomotor and default mode, respectively, while serotonin is associated with the opposite (Conio et al., 2020). Other studies have found that neurotransmitter effects on functional connectivity are connected to regional differences in excitatory-inhibitory receptor and hormone ratios (van den Heuvel et al., 2016). Gonadal hormones are increasingly recognized as critical neuromodulators of learning and memory (Taxier et al., 2020), and can pass through the blood-brain barrier and are known to impact brain function, including memory and anxiety-level behavior (Moraga-Amaro, van Waarde, Doorduin, & de Vries, 2018). These observations, combined with the fact that edge time series are mathematically exact decompositions of functional connectivity into time-varying contributions, opens the possibility for reproductive hormone to impact patterns of connectivity across time.

### Relationship With Results From Previous Analyses of Same Data

Notably, other studies have analyzed the 28andMe dataset but obtained dissimilar results. For instance, Pritschet et al. (2020) identified a strong link between the default mode network and estradiol. In contrast, we find evidence linking a specific network state to gonadotropins, FSH and LH. An important question is why these results diverge. There are a number of possible explanations, although the two most obvious deal with the timescales at which networks are modeled and characterized and the measure used to assess connectivity. Here, we analyze networks estimated using edge time series, which resolves cofluctuations at a timescale of single frames (Esfahlani et al., 2020). Previous studies, on the other hand, have focused on either static connectivity (Pritschet et al., 2020) or time-varying estimates of connectivity using sliding windows (Mueller et al., 2021). Although sliding windows resolve time-varying estimates of connectivity, the windowing procedure necessarily induces “blurring,” making it impossible to precisely temporally localize networks (Jo et al., 2021). Additionally, edge time series estimate connectivity using a correlation-based metric, which can result in opposed co-fluctuations (negative weights in a connectivity matrix), whereas previous studies used wavelet-based coherence to estimate interregional connectivity, which is bound to the interval [0, 1]. These observations underscore a more general trend in the network neuroscience community, wherein the widespread use of bespoke processing and analysis tools make it challenging to directly compare or reproduce findings. Future work must focus on standardizing these practices.

### Future Directions

The results of our study present several opportunities for future investigations. First, while we identify a putative link between high-amplitude co-fluctuations and female reproductive hormones, its implications for cognitive and clinical processes have not been fully explored. For instance, the studies from which the data originated also collected behavioral data on the participant’s level of stress, sleep, and affect across the menstrual cycle. Future studies should investigate the role of brain-hormone coupling in mediating behavioral effects.

To our knowledge, this study is the first to our knowledge to identify a significant relationship between luteinizing hormone and daily changes in high-amplitude co-fluctuations in brain connectivity across the menstrual cycle. Many studies have demonstrated luteinizing hormone’s negative association with cognition, and increased luteinizing hormone in females after menopause is associated with poorer cognition and a higher risk of dementia (Bhatta, Blair, & Casadesus, 2018; Blair et al., 2016). Future studies should examine how luteinizing hormone’s modulatory effects on cognition relate to daily changes in brain network organization and connectivity in both premenopausal and postmenopausal females.

While the dense-sampling framework allows for detailed analyses of single individuals or small cohorts of individuals (Braga & Buckner, 2017; Gordon et al., 2017; Laumann et al., 2015), its design makes generalizing to larger and more variable populations challenging. Future studies should analyze relationships between network organization, hormone concentrations, and cognition within subjects across many days of their cycle and between subjects in different endocrine states. Variability in sex hormone production occurs across the female life-span, starting at the onset of puberty and continuing across the reproductive cycle, pregnancy, and the menopausal transition. Targeting these major neuroendocrine transition states could yield greater insight into sex steroid hormones’ influence on the brain’s network architecture, mood, and cognition. Throughout the life course, changes in women’s reproductive status (e.g., puberty, use of hormonal contraceptives, pregnancy/postpartum, and perimenopause) have been associated with increased risk for mood disturbance including major depressive disorder. Yet, the neurobiological pathways by which endocrine changes give rise to depressive symptoms in some women, but not others, is unclear. A more detailed understanding of how sex hormone fluctuations produce rapid changes in brain network structure could provide a framework for understanding risk and resilience to mood disorders.

An interesting question that could be addressed in future work concerns what properties of the fMRI BOLD signal itself contribute to events. One likely contributor is the correlation structure of BOLD data. Several studies have shown that strong modular correlation structure supports high-amplitude events (Ladwig et al., 2022; Novelli & Razi, 2022; Pope et al., 2021). Another possibility is signal to noise ratio. In R. F. Betzel, Chumin, Esfahlani, Tanner, and Faskowitz (2022), the authors studied event structure in different brain systems, for example, default mode, visual, somatomotor, and so forth, including subcortical areas that have poor signal-to-noise ratio relative to neocortex. The authors found that events occurred less frequently in subcortical systems than cortex, supporting the hypothesis that overall signal quality plays a role in event generation.

An important question that arises from this paper is whether having more or fewer events on any given day (or even in a different dataset) is necessarily a “good” or “bad” thing. In general, this question should be addressed through subsequent studies that explicitly seek to relate event frequencies with other phenotypes and behavioral data. This, however, is precisely the advantage of edge time series. They provide a temporal dimension absent in the analysis of static FC through which brain network data can be studied. There are many trajectories—time-varying patterns of activity and connectivity—that lead to the same correlation structure, but are lost when we analyze static FC. Edge time series offers a window into the statistics of time-varying brain data, and possibly exposing new features.

### Limitations

Here, we analyzed fMRI data from two studies of a single individual. Although these two studies provide ample information about daily changes in brain activity and hormone levels, the single-subject nature of the study limits the generalizability of our results. It is possible that the relationships between community organization and hormone concentrations observed in this study are a result of individual processes in the subject that are not present in most of the population. Further deep-phenotyping studies with larger cohorts should be conducted to determine the generalizability of the results across the general population. This, however, belies a broader limitation. With more studies adopting a dense-sampling design, it becomes necessary to assess how much data is necessary to assess an effect of a given size. While the expectation is that having lots of data from a single brain will improve statistical power, the magnitude of this improvement remains untested or, at the very least, not well documented. Even basic questions concerning the typical size of effects in dense-sampling studies have not been fully reported—for example, the extent to which fluctuations in hormone levels are coupled with brain-based variables. Thus, power estimates for these types of studies are lacking and should be the focus of future research.

A second limitation concerns the detection and characterization of edge time series and high-amplitude co-fluctuations. Recent studies have suggested that some of their properties can be anticipated from the static, that is, time-invariant, functional connectivity matrix alone (Novelli & Razi, 2022), downplaying their interpretation as dynamic events. Determining the features of higher order network constructs like edge time series and edge connectivity remains an active area of research (R. F. Betzel, Cutts, et al., 2022; Cooper et al., 2021; Esfahlani et al., 2022). Additional studies are necessary to address this and related open questions.

We note, however, that while our findings suggest a hormonal contribution to high-amplitude events, other factors likely also play important roles, including the underlying anatomical connectivity, whose network organization shapes the spatial topography of event co-fluctuations (Pope et al., 2021). Future work should be directed to tease apart the contributions of structural connectivity (Seider et al., 2021) as well as other factors, including those molecular, vascular (Bright, Whittaker, Driver, & Murphy, 2020), and hemodynamic (Chang & Glover, 2009).

An additional limitation concerns the dynamic interpretation of edge time series. Here, we use edge time series to track frame-by-frame changes in network structure, detect high-amplitude frames, and partition those frames into repeating clusters. In this way, we use edge time series, not a means of studying brain dynamics per se, but to decompose FC and filter time points. Indeed, there remains ongoing debate, both in the edge time series literature (see Novelli & Razi, 2022), but also in the time-varying FC literature (see Laumann et al., 2017; Liégeois et al., 2017) concerning the extent to which brain activity and connectivity are truly dynamic and marked by distinct changes in underlying correlation structure. While edge time series do not require the parameterizations that sliding windows do (these may make detecting “true” dynamics difficult), it remains unclear whether the fluctuations observed in edge time series are, indeed, evidence of such dynamics. This remains an important area for future research (Lurie et al., 2020).

We note, however, there are some important differences between our study and those previous studies. In particular, our study focuses on rapid, that is, framewise, changes in co-fluctuation patterns. Even in sliding-window analyses, access to this timescale is limited and the networks resulting from each technique exhibit dissimilar topological profiles (Esfahlani et al., 2022). In addition, our study focused on gonadotropic *and* ovarian hormones, and focused not on distinct phases of the menstrual cycle or on peaks in serum levels, but rather on day-to-day fluctuations in hormone levels.

Here, we focus on endogenous hormones only. However, exogenous hormones—introduced through the oral contraceptive—could, in principle, also be related to network connectivity and brain states. However, we note that the serum concentrations of the exogenous hormones ethinyl estradiol and levonorgestrel are very low and, consistent with previous studies (Mueller et al., 2021; Pritschet et al., 2020), were not examined here. Future studies should investigate the interplay of exogenous and endogenous hormones and their contributions to time-varying connectivity.

Another potential concern is related to pooling data from across two experiments. In principle, this decision was made to enhance statistical power—it doubles the number of samples. However, the data from each experiment was collected under different conditions (with versus without oral contraceptive), raising concerns that the datasets are too dissimilar to warrant aggregating. As a safeguard against the possibility that our results are biased by the pooling procedure, we performed a number of supplementary analyses. Most importantly, we verified that the main result—an association between events and FSH/LH concentrations—reproduces when we analyzed either experimental dataset in isolation, that is, without pooling. A partial explanation for this replication is that neither FSH nor LH exhibited significant differences in mean concentration between experiments (see Supporting Information Figure S5). Another explanation for the replication is that, rather than using product-moment correlations to link events and hormones—a measure that is sensitive to amplitude changes and can be driven by outliers or spikes in data—we used rank correlations which transforms data to an ordinal scale before calculating correlations. Collectively, these and other efforts help reduce the likelihood that the reported results are driven by differences in experimental conditions.

In general, we find evidence that hormones fluctuate from day to day and that the structure of these fluctuations are preserved, irrespective of whether or not the subject took an oral contraceptive (see Supporting Information Figure S20). Though the causes of the fluctuations are not always clear, they likely include lifestyle factors—for example, diet, sleep, and so forth—and in the case of experiment 2, the effects of an oral contraceptive. Consider a “ground truth” where the number of events observed on any scan session was proportional to hormone concentration plus some random noise. Were this the case, then we would expect that any fluctuation in hormones, irrespective of the cause, would yield a proportional change in the number of events. Indeed, we find evidence that this is the case and that, at least for FSH and LH, the relationship between events and hormone concentration is not interrupted by oral contraceptives. However, they cannot conclusively rule out the possibility that there exist experiment-dependent relationships between brain networks and hormone concentrations that would appear with additional data from the same individual or by expanding the scope of data collection to include more individuals. These two directions represent potentially fruitful follow-ups to the present study.

A final limitation concerns our interpretation of endocrine effects on recorded brain activity and connectivity. Here, we find that hormone concentrations are associated with high-amplitude co-fluctuations that have been shown to shape whole-brain patterns of functional connectivity (R. F. Betzel, Cutts, et al., 2022; Esfahlani et al., 2020, 2022; Pope et al., 2021; Sporns et al., 2021). However, hormones also impact brain vasculature (Krause, Duckles, & Pelligrino, 2006). Because the fMRI BOLD signal is an indirect measure of brain activity that depends critically on neurovascular coupling, an alternative explanation is that true effect of gonadotropins is on brain vasculature, which modulates the hemodynamic response. Future, and more targeted, studies should aim to disentangle these effects.

### Conclusions

In conclusion, our study posits a link between high-amplitude, network-level co-fluctuations and the human endocrine system. Specifically, we report an association between the frequency of dynamic network states and variation in luteinizing and follicle-stimulating hormones. Our work addresses questions concerning the factors contributing to high-amplitude co-fluctuations while opening up new opportunities for future studies.

## MATERIALS AND METHODS

### Datasets

Neuroimaging and endocrine data comes from a single subject (author L.P.) scanned over a course of 30 days, on two separate occasions (Study 1 and Study 2). The subject had no history of neuropsychiatric diagnosis, endocrine disorders, or prior head trauma and no history of smoking. She had a history of regular menstrual cycles (no missed periods, cycle occurring every 26–28 days). In the 12 months prior to the first 30-day data collection period, the subject was free from hormone-based medication. In the second study, the participant was on a hormone regimen (0.02 mg ethinyl-estradiol, 0.1 mg levonorgestrel, Aubra, Afaxys Pharmaceuticals), which she began 10 months prior to the start of data collection. The pharmacological regimen used in Study 2 chronically and selectively suppressed progesterone while leaving estradiol dynamics largely indistinguishable from Study 1. The participant gave written informed consent and the study was approved by the University of California, Santa Barbara, Human Subjects Committee.

Both studies consisted of similar design and included components that were analyzed here. Test sessions began with a daily questionnaire (assessments of stress, sleep, anxiety, and affective state), followed by a spatial navigation task (not reported here). Time-locked collection of serum and whole blood started each day at 10:00 am in Study 1 and 11:00 am in Study 2 (±30 min). Endocrine samples were collected, at minimum, after 2 hours of no food or drink consumption (excluding water). Additionally, the participant abstained from caffeinated beverages prior to test sessions. Following behavioral assessment, the participant underwent a 1-hour MRI scan session that included the acquisition of structural and functional images.

Neuroimaging data was collected on a Siemens 3T Prisma scanner with a 64-channel phased-array head coil. Scans were collected around the same time each day (11:00 am local time). For each scanning session, a high-resolution T1-weighted anatomical sequence (MPRAGE) was acquired (TR = 2,500 ms, TE = 2.31 ms, T_1_ = 934 ms, flip angle = 7°). Following this, a 10-minute resting-state sequence was acquired, using a T_2_*-weighted multiband echo-planar (EPI) sequence was acquired (72 oblique slices, TR = 720 ms, TE = 37 ms, voxel size 2 mm^3^, flip angle = 52°, multiband factor = 8). To mitigate against motion, a custom 3D-printed foam head case was employed for days 8–30 of the first 30-day period, and for days 1–30 of the second 30-day period.

### Endocrine Procedures

A licensed phlebotomist inserted a saline-lock intravenous line into the dominant or nondominant hand or forearm daily to evaluate hypothalamic-pituitary-gonadal axis hormones, including serum levels of gonadal hormones (17*β*-estradiol, progesterone, and testosterone) and the pituitary gonadotropins luteinizing hormone (LH) and follicle stimulating hormone (FSH). One 10-cc-mL blood sample was collected in vacutainer SST (BD Diagnostic Systems) each session. The sample clotted at room temperature for 45 minutes until centrifugation (2,000 × g for 10 minutes) and were then aliquoted into three 1 mL microtubes. Serum samples were stored at −20° until assayed. Serum concentrations were determined via liquid chromatography mass spectrometry (for all steroid hormones) and immunoassay (for all gonadotropins) at the Brigham and Women’s Hospital Research Assay Core. Assay sensitivities, dynamic range, and intra-assay coefficients of variation (respectively) were as follows: estradiol 1 pg/mL, 1–500 pg/mL, <5% relative standard deviation (RSD), 0.05 ng/mL, 0.05–10 ng/mL, 9.33% RSD; testosterone, 1.0 ng/dL 1–2,000 ng/dL, <4% RSD. FSH and LH levels were determined via chemiluminescent assay (Beckman Coulter). The assay sensitivity, dynamic range, and intra-assay coefficient of variation were as follows: FSH, 0.2 mlU/mL, 0.2–200 mIU/mL, 3.1–4.3%; LH, 0.2 mIU/mL, 0.2–250 mIU/mL, 4.3–6.4%. Importantly, we note that LC-MS assessments of exogenous hormone concentrations (attributable to the hormone regime itself) showed that serum concentrations of ethinyl estradiol were very low (*M* = 0.01 ng/mL; range 0.001–0.016 ng/mL) and below 1.5 ng/mL for levonorgestrel (*M* = 0.91 ng/mL; range = 0.03–1.45 ng/mL). This ensures that the brain hormone associations reported in Study 2 are still due to endogenous estradiol action.

Preprocessing was performed using *fMRIPrep* (20.2.0) (Esteban et al., 2018), which is based on Nipype 1.5.1 (Gorgolewski et al., 2011). The following description of fMRIPrep’s preprocessing is based on boilerplate distributed with the software covered by a ‘no rights reserved’ (CCO) license. Internal operations of fMRIPrep use Nilearn 0.6.2 (Abraham et al., 2014), mostly within the functional processing workflow. The preprocessing pipelines employ functions from ANTs (2.3.3), FreeSurfer (6.0.1), FSL (5.0.9), and AFNI (20160207).

T1-weighted (T1w) images were corrected for intensity nonuniformity (INU) with N4BiasFieldCorrection (Tustison et al., 2010), distributed with ANTs (Avants, Epstein, Grossman, & Gee, 2008), and used as T1w-reference throughout the workflow. The T1w-reference was then skull-stripped with a Nipype implementation of the antsBrainExtraction.sh workflow, using NKI as target template. Brain tissue segmentation of cerebrospinal fluid (CSF), white matter (WM), and gray matter (GM) was performed on the brain-extracted T1w using fast (Zhang, Brady, & Smith, 2001). Brain surfaces were reconstructed using recon-all (Dale, Fischl, & Sereno, 1999), and the brain mask estimated previously was refined with a custom variation of the method to reconcile ANTs-derived and FreeSurfer-derived segmentations of the cortical gray matter of Mindboggle (Klein et al., 2017). Volume-based spatial normalization to one standard space (MNI152NLin2009cAsym) was performed through nonlinear registration with antsRegistration, using brain-extracted versions of both T1w reference and the T1w template. The following template was selected for spatial normalization: ICBM 152 Nonlinear Asymmetrical template version 2009c (Fonov, Evans, McKinstry, Almli, & Collins, 2009). For each of the BOLD runs found per subject (across all tasks and sessions), the following preprocessing was performed. First, a reference volume and its skull-stripped version were generated using a custom methodology of *fMRIPrep*. Susceptibility distortion correction (SDC) was omitted. The BOLD reference was then co-registered to the T1w reference using bbregister (FreeSurfer), which implements boundary-based registration (Greve & Fischl, 2009). Co-registration was configured with six degrees of freedom. Head-motion parameters with respect to the BOLD reference (transformation matrices and six corresponding rotation and translation parameters) are estimated before any spatiotemporal filtering using mcflirt (Jenkinson, Bannister, Brady, & Smith, 2002). BOLD runs were slice-time corrected using 3dTshift from AFNI (Cox & Hyde, 1997). The BOLD time series (including slice timing correction when applied) were resampled onto their original, native space by applying the transforms to correct for head motion. These resampled BOLD time series will be referred to as *preprocessed BOLD in original space*, or just *preprocessed BOLD*. The BOLD time series were resampled into standard space, generating a *preprocessed BOLD run in MNI152NLin2009cAsym space*. First, a reference volume and its skull-stripped version were generated using a custom methodology of fMRIPrep. Several confounding time series were calculated based on the preprocessed BOLD: framewise displacement (FD), DVARS and three region-wise global signals. FD was computed using two formulations following Power (absolute sum of relative motions; Power et al., 2014) and Jenkinson (relative root mean square displacement between affines; Jenkinson et al., 2002). FD and DVARS are calculated for each functional run, both using their implementations in Nipype following the definitions by Power et al. (2014). The three global signals are extracted within the CSF, the WM, and the whole-brain masks. Additionally, a set of physiological regressors were extracted to allow for component-based noise correction (Behzadi, Restom, Liau, & Liu, 2007). Principal components are estimated after high-pass filtering the *preprocessed BOLD* time series (using a discrete cosine filter with 128-s cutoff) for the two *CompCor* variants: temporal (tCompCor) and anatomical (aCompCor). tCompCor components are then calculated from the top 2% variable voxels within the brain mask. For aCompCor, three probabilistic masks (CSF, WM, and combined CSF + WM) are generated in anatomical space. The implementation differs from that of Behzadi et al. in that instead of eroding the masks by two pixels on BOLD space, the aCompCor masks are subtracted a mask of pixels that likely contain a volume fraction of GM. This mask is obtained by dilating a GM mask extracted from the FreeSurfer’s *aseg* segmentation, and it ensures components are not extracted from voxels containing a minimal fraction of GM. Finally, these masks are resampled into BOLD space and binarized by thresholding at 0.99 (as in the original implementation). Components are also calculated separately within the WM and CSF masks. For each CompCor decomposition, the *k* components with the largest singular values are retained, such that the retained components’ time series are sufficient to explain 50 percent of variance across the nuisance mask (CSF, WM, combined, or temporal). The remaining components are dropped from consideration. The head-motion estimates calculated in the correction step were also placed within the corresponding confounds file. The confound time series derived from head motion estimates and global signals were expanded with the inclusion of temporal derivatives and quadratic terms for each (Satterthwaite et al., 2013). Frames that exceeded a threshold of 0.5 mm FD or 1.5 standardized DVARS were annotated as motion outliers. All resamplings can be performed with a single interpolation step by composing all the pertinent transformations (i.e., head-motion transform matrices, susceptibility distortion correction when available, and co-registrations to anatomical and output spaces). Gridded (volumetric) resamplings were performed using antsApplyTransforms, configured with Lanczos interpolation to minimize the smoothing effects of other kernels (Lanczos, 1964). Nongridded (surface) resamplings were performed using mri_vol2surf (FreeSurfer).

The Schaefer parcellation (Schaefer et al., 2018) was used to delineate 400 regions on the cortical surface. To transfer the parcellation from *fsaverage* to *subject* space, FreeSurfer’s mris_ca_label function was used in conjunction with a pretrained Gaussian classifier surface atlas (Fischl et al., 2004) to register cortical surfaces based on individual curvature and sulcal patterns. The result is a volumetric parcellation rendered in subject anatomical space that follows the cortical ribbon estimated via FreeSurfer’s recon-all process.

Each preprocessed BOLD image was linearly detrended, band-pass filtered (0.008–0.08 Hz), confound regressed and standardized using Nilearn’s signal.clean function, which removes confounds orthogonally to the temporal filters. The confound regression strategy included six motion estimates, mean signal from a white matter, cerebrospinal fluid, and whole brain mask, derivatives of these previous nine regressors, and squares of these 18 terms (Satterthwaite et al., 2013). Spike regressors were not applied. The 36 parameter strategy (with and without spike regression) has been show to be a relatively effective option to reduce motion-related artifacts (Parkes, Fulcher, Yücel, & Fornito, 2018). An alternative preprocessesing strategy was also employed to evaluate the stability of the findings. This strategy included six motion estimates, derivatives of these previous six regressors, and squares of these 12 terms, in addition to five anatomical CompCor components (Behzadi et al., 2007). Following these preprocessing operations, the mean signal was taken at each node in volumetric anatomical space.

### Edge Time Series

Let *z*_*i*_ = [*z*_*i*_(1), … *z*_*i*_(*T*)] be the z-scored time series for region *i*. Most network neuroscience analyses define the functional connectivity between pairs of regions {*i*, *j*} as the Pearson correlation of their activity, that is, *r*_*ij*_ = $\frac{1}{T-1}$ ∑_*t*_ *z*_*i*_(*t*) · *z*_*j*_(*t*).

Recently, we proposed a “temporal unwrapping” of functional connection weights by omitting the summation. That is, we calculate the instantaneous magnitude and sign of co-fluctuation between pairs of brain regions as *e*_*ij*_(*t*) = *z*_*i*_(*t*) · *z*_*j*_(*t*) (R. F. Betzel, Cutts, et al., 2022; Esfahlani et al., 2020; Faskowitz et al., 2020). This approach has the benefit of resolving changes in pairwise interactions at a temporal resolution of single frames. It also is deeply related to the Pearson correlation and functional connectivity—the temporal average of a region pairs’ edge time series is exactly equal to its connection weight. In this way, edge time series can be viewed as precise temporal decompositions of functional connectivity. In this study, we calculated edge time series for every pair of cortical and subcortical regions for every scan session.

### Event Detection

The brainwide level of network activity at any instant can be summarized as the root sum of square over all edges co-fluctuations:

$$RSS\left(t\right)=\sum_{i,j\neq i}e_{ij}\left(t\right).$$ (1)

In previous studies we demonstrated that *RSS* exhibits “bursty” behavior, such that most time points express low-amplitude co-fluctuations while a relatively small number exhibit large *RSS* values (R. F. Betzel, Cutts, et al., 2022). These “events” are thought to reflect underlying anatomical connectivity (Pope et al., 2021), are highly individualized (R. F. Betzel, Cutts, et al., 2022), and contribute disproportionately to the time-averaged pattern of functional connectivity (Esfahlani et al., 2020; Sporns et al., 2021).

Recently, we proposed a simple statistical test for detecting events. This test works by comparing observed *RSS* values with those generated under a temporal null model in which regional time series are circularly shifted by a random offset in either direction. This procedure generates a null distribution of *RSS* values against which an empirical value can be compared statistically. For each frame we estimated a nonparametric *p* value by counting the fraction of null *RSS* values that exceeded the observed value. We compared *p* values against an adjusted critical value while fixing the false discovery rate at *q* = 0.05 (Benjamini & Hochberg, 1995). Note that this null model necessarily destroys the correlation structure (functional connectivity) and can be viewed as a principled method for selecting a threshold for events.

Once a statistical threshold for events is determined, we identify temporal contiguous sequences of suprathreshold frames, which we refer to as *event segments*. We discard any such segments that include a high-motion frame or are within two frames of a high-motion frame and, from those segments, extract as a representative co-fluctuation pattern the frame corresponding to the peak *RSS*. We repeat this procedure for all scans, retaining the peak co-fluctuation patterns.

### Cluster Definition

In previous studies, we demonstrated that high-amplitude co-fluctuation patterns can be clustered into a series of states (R. F. Betzel, Cutts, et al., 2022). To do this, we compute the similarity (correlation) between all pairs co-fluctuation patterns extracted during event segments. This results in a pattern × pattern matrix, which we submitted to a generalized version of the Louvain algorithm (Blondel, Guillaume, Lambiotte, & Lefebvre, 2008; Jutla, Jeub, & Mucha, 2011) for modularity maximization algorithm (Newman & Girvan, 2004). Modularity maximization is a computational heuristic for detecting community structure in networked data. It defines communities (clusters) as groups elements whose internal density of connections maximally exceed what would be expected. In this context, we defined the expected weight of connections to be equal to the mean similarity between all pairs of patterns.

Modularity maximization with the Louvain algorithm is nondeterministic and, depending upon initial conditions, can yield dissimilar results. Accordingly, we ran the algorithm 1,000 times with different random seeds. We resolved variability across these different seeds using a consensus clustering algorithm in which we iteratively clustered the module co-assignment matrix until convergence (see any of R. F. Betzel, Cutts, et al., 2022; R. F. Betzel, 2020; R. F. Betzel et al., 2017; for details of this algorithm). The resulting consensus partition assigned each co-fluctuation pattern to nonoverlapping clusters.

## ACKNOWLEDGMENTS

We thank Haily Merritt and Jacob Tanner for providing feedback on an early version of this manuscript.

## SUPPORTING INFORMATION

Supporting information for this article is available at https://doi.org/10.1162/netn_a_00307.

## AUTHOR CONTRIBUTIONS

Sarah Greenwell: Formal analysis; Investigation; Methodology; Software; Visualization; Writing – original draft; Writing – review & editing. Joshua Faskowitz: Data curation; Resources; Software; Writing – review & editing. Laura Pritschet: Conceptualization; Data curation; Methodology; Resources; Writing – review & editing. Tyler Santander: Conceptualization; Data curation; Methodology; Resources; Writing – review & editing. Emily Jacobs: Conceptualization; Data curation; Methodology; Resources; Writing – review & editing. Richard Betzel: Conceptualization; Data curation; Investigation; Methodology; Project administration; Resources; Software; Supervision; Validation; Visualization; Writing – original draft; Writing – review & editing.

## FUNDING INFORMATION

Richard Betzel, National Science Foundation (https://dx.doi.org/10.13039/501100008982), Award ID: 2023985.

## TECHNICAL TERMS

Edge or co-fluctuation time series: The element-wise product of two parcel time series; the average value of an edge time series is exactly the functional connectivity weight.
Event: Frames when many edge time series exhibit greater-than-expected amplitudes; detected statistically by comparing the global amplitude of edge time series (root sum of squared values) with a temporal null model.
Functional connectivity: Operationalized as the correlation coefficient between two parcel time series.
Node time series: The mean fMRI BOLD activity for a region of interest, network node, or atlas parcel.
Event community, cluster, or state: After detecting events, we can group them into clusters using data-driven algorithms and based on their spatial similarity to one another.
