# Supplementary material for: High-amplitude network co-fluctuations linked to variation in hormone concentrations over the menstrual cycle
Source: Netw Neurosci. 2023 Oct 1;7(3):1181–205. doi: 10.1162/netn_a_00307 (PMC10473261; doi:10.1162/netn_a_00307)
Supplement: Supplementary file 1 [file netn-7-3-1181-s001.pdf]

Greenwell, S., Faskowitz, J., Pritschet, L., Santander, T., Jacobs, E. G. & Betzel, R. E. (2023). Supporting information for "High-amplitude network co-fluctuations linked to variation in hormone concentrations over the menstrual cycle." *Network Neuroscience*. Advance publication. https://doi.org/10.1162/netn_a_00307

Journal: **NETWORK NEUROSCIENCE**

RESEARCH

Supplementary Material to "High-amplitude network co-fluctuations linked to variation in hormone concentrations over the menstrual cycle"

Sarah Greenwell[1], Joshua Faskowitz[1,2], Laura Pritschet[3], Tyler Santander[3], Emily G. Jacobs[3,4], and Richard F. Betzel[1,2,5,6]

[1]Department of Psychological and Brain Sciences, Indiana University, Bloomington, IN 47405

[2]Program in Neurosciences, Indiana University, Bloomington, IN 47405

[3]Department of Psychological and Brain Sciences, University of California, Santa Barbara, Santa Barbara, CA 93106

[4]Neuroscience Research Institute, University of California, Santa Barbara, Santa Barbara, CA 93106

[5]Cognitive Science Program, Indiana University, Bloomington, IN 47405

[6]Network Science Institute, Indiana University, Bloomington, IN 47405

Keywords: [Edge-centric, functional connectivity, time-varying networks]

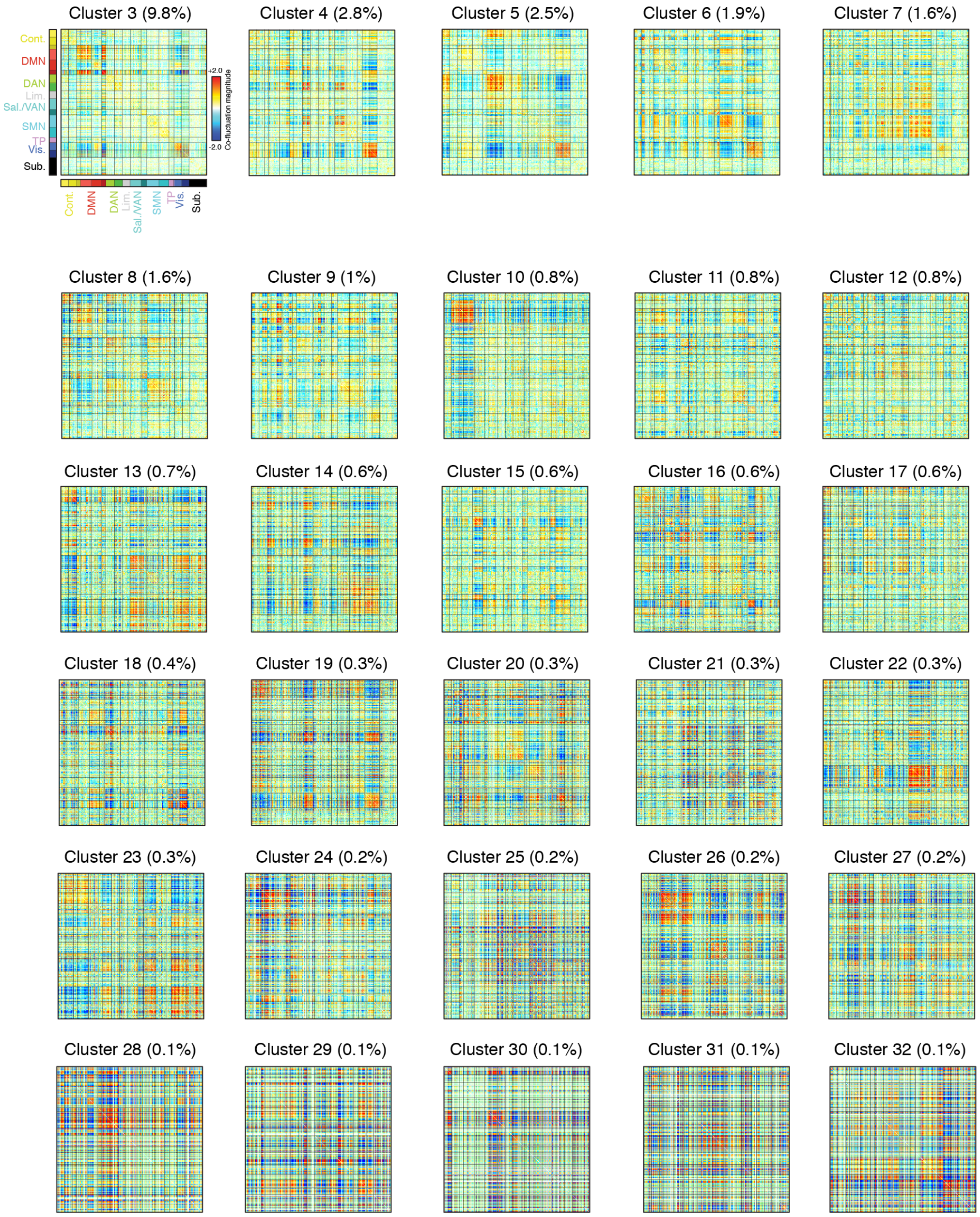

Figure S1. Centroids for remaining co-fluctuation communities. In the main text we clustered "event" co-fluctuation patterns and analyzed the top two communities by frequency. Here, we show the centroids for the remaining 30 communities.

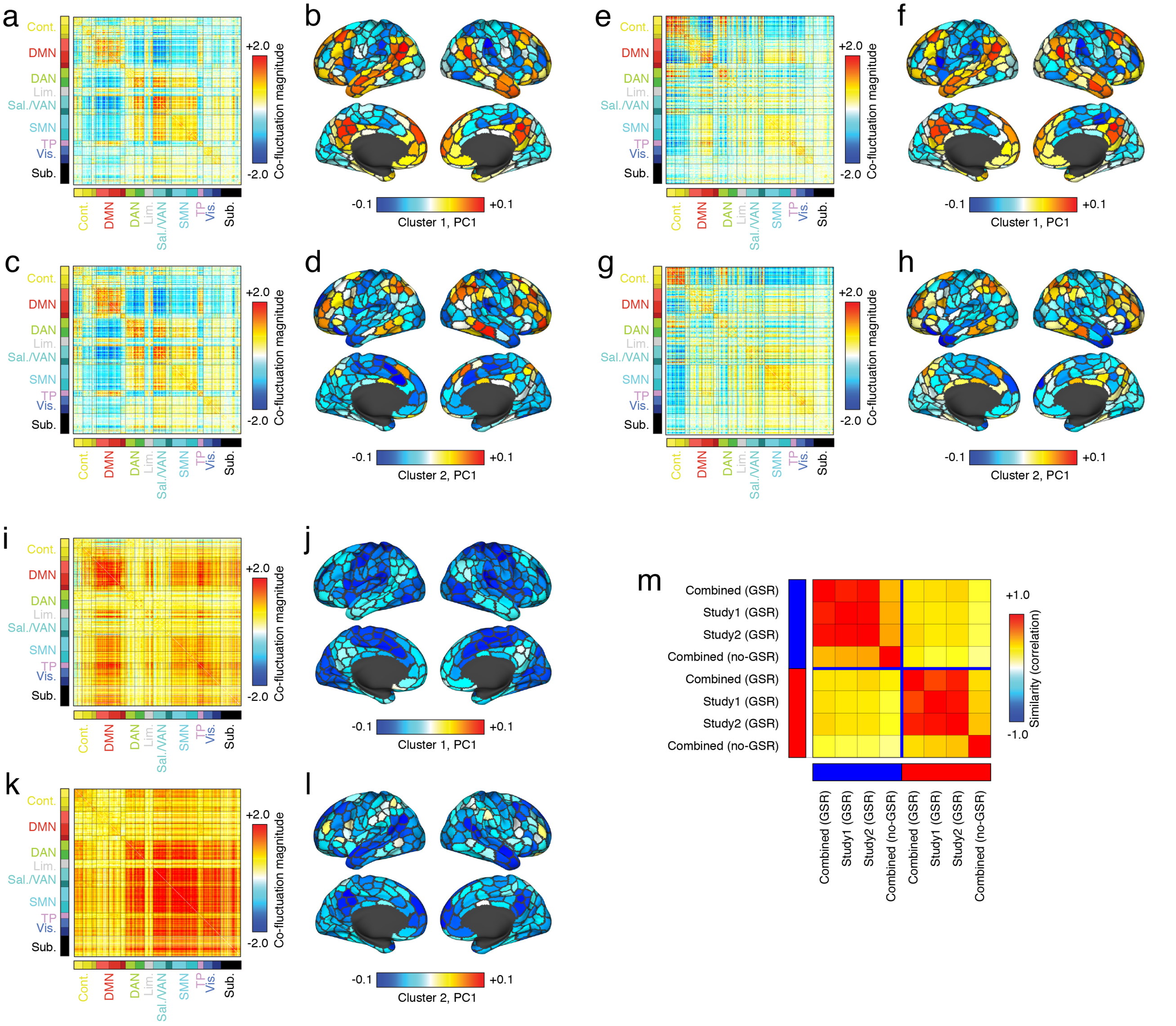

Figure S2. Robustness of communities 1 and 2 to processing decisions. In the main text we clustered "event" co-fluctuation patterns and analyzed the top two communities by frequency. To demonstrate the robustness of these two communities, we repeated this procedure, by splitting the 60 scan sessions into their two respective experiments (Study 1 and Study 2; panels *a-d* and *e-h*, respectively). We also analyzed data processed without global signal regression (panels *i-l*). In each quartet of panels, we highlight the top two communities by frequency. In panel *m*, we show the similarity of community centroids to one another. In general, we find that when splitting data by experiment, we maintain an excellent correspondence with the original data. We also find a strong correspondence between the original data and data processed without global signal regression.

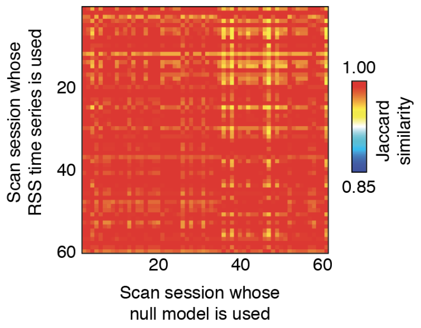

Figure S3. Effect of null model on detected events. In the main text, we analyzed co-fluctuation patterns estimated from resting-state time series by comparing the global RMS time series against a null distribution generated using a circular shift null model. For each scan, we considered a null distribution generated using the empirical data from that scan. Here, we test the effect of using null distributions from all other 59 scans on the event structure. Specifically, we compared the binary mask of statistically significant frames (reported in main text) with masks generated using null models from the remaining 59 scans. As a measure of similarity we used the Jaccard similarity measure (bounded between 0 and 1, i.e. minimal and maximal overlap between significant frames). Here, we show that irrespective of the scan session in which the null model was generated, the significant frames vary only slight (note that the colormap varies over a truncated range from 0.85 to 1.00).

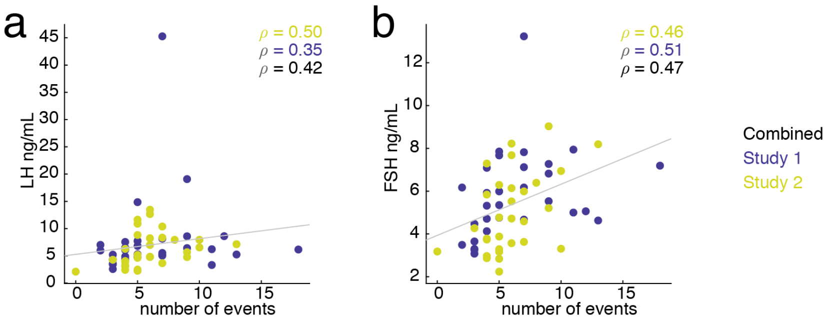

Figure S4. Robustness of correlation between state frequency and gonadotropins. In the main text we reported correlations between gonadotropin concentration and the frequency (counts) of community (brain state) 1 with luteinizing and follicle-stimulating hormone. Here, we repeat the analysis after splitting the data by experiment (Study 1 *versus* Study 2). (*a*) Scatterplot of community frequency and luteinizing hormone (black is combined correlation, yellow is Study 1, blue is Study 2). (*b*) Analogous plot, but for follicle-stimulating hormone.

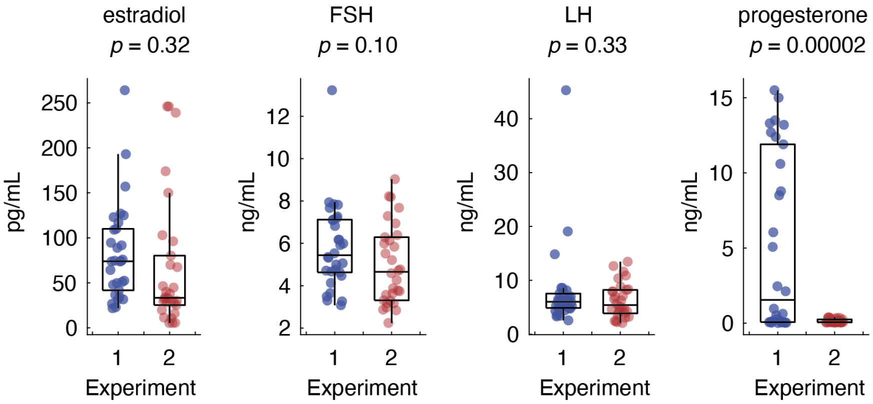

Figure S5. Comparison of hormone concentrations between experiments 1 and 2. All reported tests are two-sample t-tests.

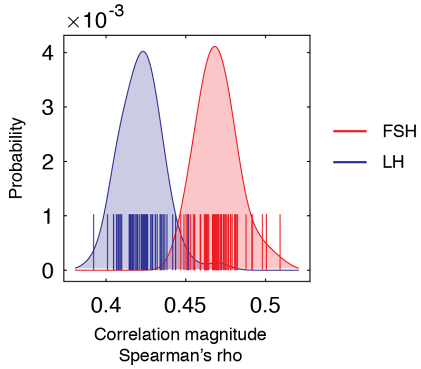

Figure S6. Impact of “leave one out” analysis on state frequency and gonadotropin correlations. Distribution of correlation coefficients after removing data from single scans and recomputing the Spearman correlation coefficient. In this plot, red corresponds follicle-stimulating hormone and blue corresponds to luteinizing hormone.

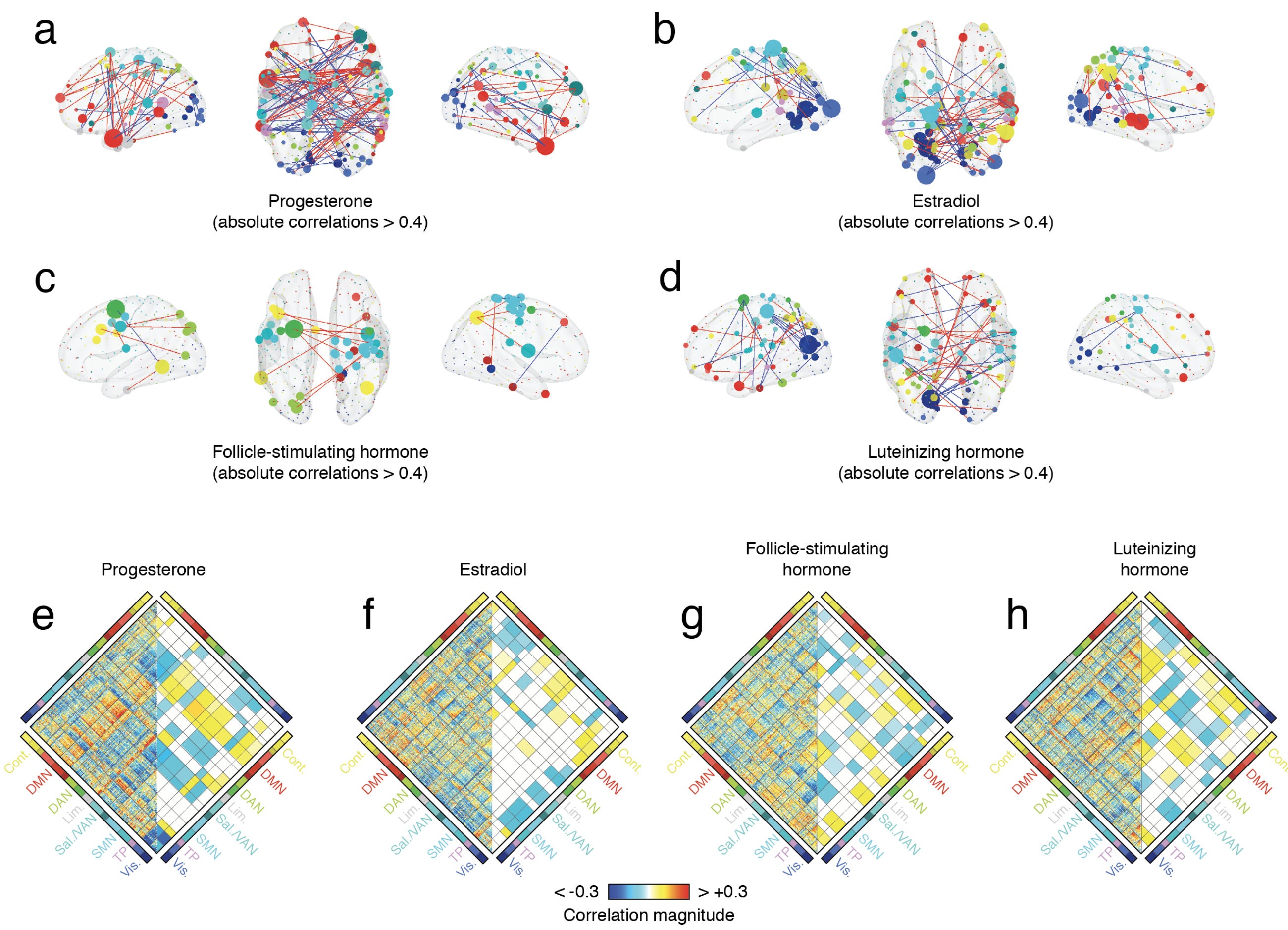

36 **Figure S7. Edge- and system-level correlations with hormone concentration for community 2.** In the main text we calculated the correlation of hormone
37 concentrations with edge-level co-fluctuation magnitudes for cluster 1. Here, we report the results of the same analysis carried out using estimates of state 2.
38 Panels *a-d* depict strongest correlations in anatomical space. Node size is proportional to the mean correlation magnitude of a node's edges. Node color was
39 determined by brain system. Edge color denotes positive (red) and blue (negative) correlations. Panels *e-h* depict full matrices of correlations (*left*) alongside
40 mean system-level correlations (*right*).

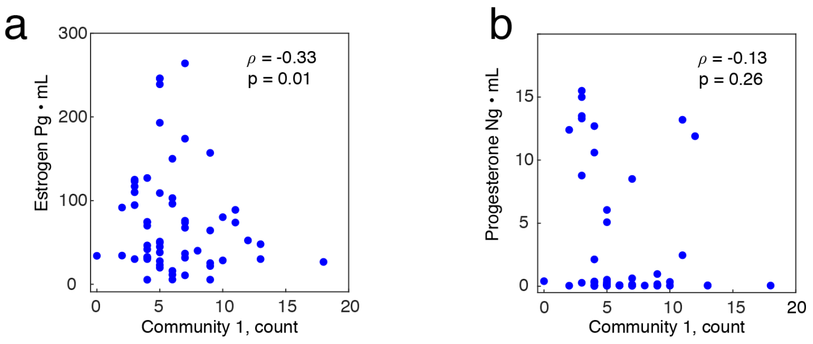

Figure S8. Correlations of sex hormone concentration with frequency of community 1. In the main text, we reported positive correlations between gonadotropic hormones and the frequency of community 1. Here, we report analogous results for sex hormones (*a*) estradiol and (*b*) progesterone. Note that the correlation with estradiol is statistically significant only without correcting for multiple comparisons.

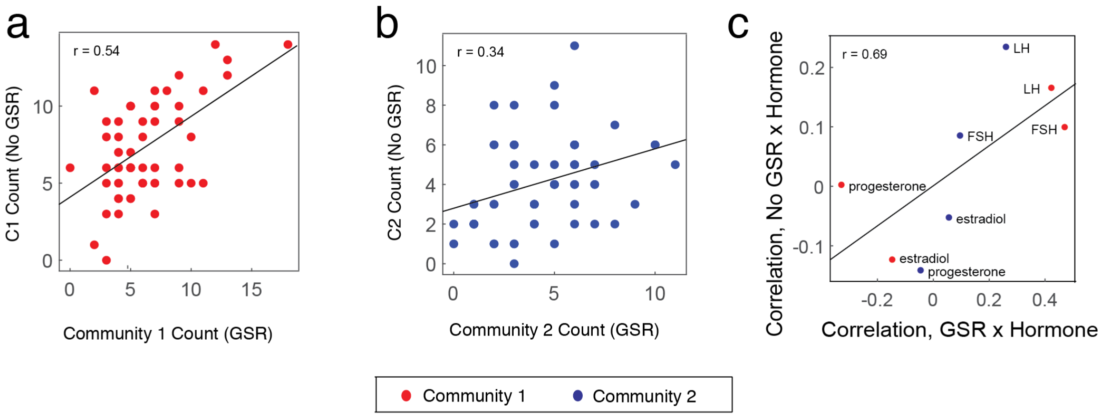

Figure S9. Effect of global signal regression on state frequency and correlations. In the main text we analyzed fMRI data that had been processed using a pipeline that included global signal regression. Here, we report results using the same data but processed without global signal regression. The analysis procedures were performed identically for both datasets. After obtaining consensus clusters, we mapped communities to those obtained following the global signal regression pipeline. Panels *a* and *b* show correlations between state frequencies (how often a given community appeared on any one scan session), for communities 1 and 2. We find a positive correspondence in both cases. In the main text we also computed the correlation between state frequency and hormone concentration. We find that without global signal regression, the overall magnitude of correlations is decrease. However, the overall pattern of correlations is largely preserved (see panel *c*).

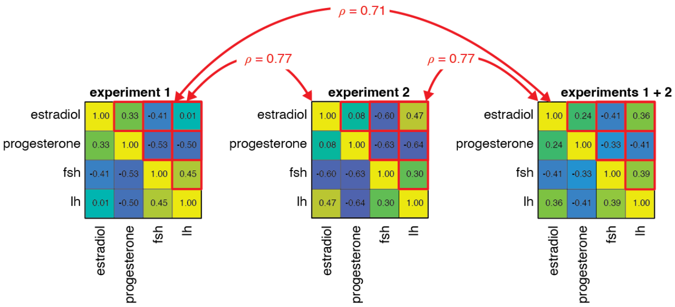

Figure S10. Similarity of hormone co-fluctuations across experiments. We calculated the (Spearman) correlations between hormone concentrations using only samples from experiment 1, experiment 2, and with both datasets combined. In general, we find that the correlation structure, which measures whether day-to-day changes in one hormone are coincident with changes in another, is remarkably stable across experimental conditions, despite differences in hormone profile induced by oral contraceptive. In this figure, we show correlation matrices for experiment 1 (left), experiment 2 (middle), and experiments 1 + 2 (right). The red arrows indicate the similarity–Spearman rank correlation–of upper triangle elements (outlined in red).
